# Regional and vertical scaling of water vapor with temperature over Japan during extreme precipitation in a changing climate

**DOI:** 10.1038/s41598-025-22287-6

**Published:** 2025-10-15

**Authors:** Sridhara Nayak, Tetsuya Takemi

**Affiliations:** 1 Research and Development Center, Japan Meteorological Corporation, 530-0011 Osaka, Japan; 2 Disaster Prevention Research Institute, Kyoto University, Kyoto, 611-0011 Japan

**Keywords:** Extreme precipitation event, Clausius-Clapeyron equation, Precipitation-temperature relationship, Water vapor, Climate sciences, Environmental sciences, Hydrology

## Abstract

Water vapor in the atmosphere plays a critical role in the occurrence of extreme precipitation events. As temperatures increase, the atmosphere can hold more water vapor, which leads to an increase in the intensity of extreme precipitation events. The Clausius-Clapeyron (CC) equation predicts that the intensity of extreme precipitation events will scale with temperature at a rate of approximately 7% per degree Celsius. However, previous studies have been limited to explore how the availability of water vapor varies at different pressure levels in the atmosphere on wet days. In our study, we found a strong positive relationship between specific humidity and temperature on wet days in the lower atmospheric levels over Japanese regions, while no significant relationship was found at higher levels. The rate of change of specific humidity with temperature on wet days varied by region, but was within the range of 8.3 ± 2.4% per degree Celsius. Our findings suggest that extreme precipitation events will become more intense in the future warming climate across all regions of Japan. This intensification is mainly driven by increased moisture availability on wet days, as well as changes in temperature and atmospheric dynamics, which are expected to influence extreme precipitation events.

**Supplementary Information:**

The online version contains supplementary material available at 10.1038/s41598-025-22287-6.

## Introduction

**Fig. 1:**
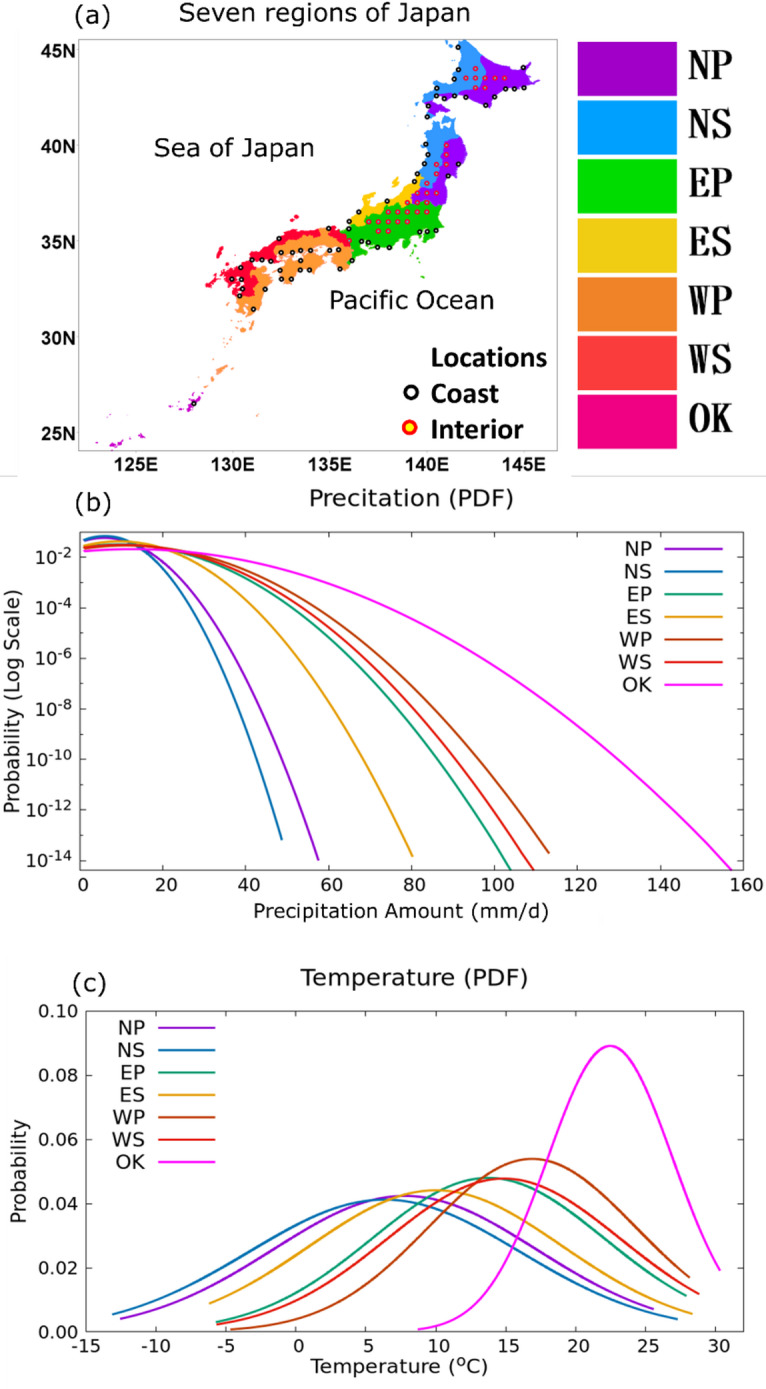
(**a**) Location map of seven regions of Japan: Sea of Japan side of North Japan (NS); Sea of Japan side of East Japan (ES); Sea of Japan side of West Japan (WS); Pacific Ocean side of North Japan (NP); Pacific Ocean side of East Japan (EP); Pacific Ocean side of West Japan (WP); and Okinawa (OK). Probability Density Functions (PDFs) of (**b**) daily precipitation and (**c**) daily mean temperature during the wet days from APHRODITE data. Black circles in (**c**) indicate the coastal locations and red circles in (**c**) represent interior locations (more than 50 km inland), used for analyzing the CC scaling over coast and interior region of Japan.

Extreme precipitation events can cause devastating floods and landslides, and many regions across the globe are increasingly vulnerable to such natural disasters^1–5^. The Clausius-Clapeyron (CC) equation predicts that for every 1 °C rise in temperature, the atmosphere can hold approximately 7% more water vapor. This means that under future climate scenarios with warmer temperatures, the intensity of extreme precipitation events is expected to increase significantly^5–10^. Observational evidence and modeling studies have confirmed that there is a CC-like scaling of extreme precipitation events with surface air temperature up to a certain threshold of approximately 25 °C^11–17^. However, in a future warming climate, the warmer atmosphere is expected to extend this relationship to a higher temperature range and may result in an increase in the intensity of extreme precipitation events^7,13,18–20^. This indicates a significant risk of water-related hazards in the future. One critical mechanism for intensifying precipitation events is the availability of sufficient water vapor in the atmosphere, as water vapor acts like a fuel to create precipitation through an efficient transport of atmospheric energy due to latent heat release^8,21–25^. Some studies have highlighted that atmospheric dew point temperature is an effective indicator of the precipitation-temperature relationship, with the most effective level being 850 hPa^26,27^. However, how water vapor at different pressure levels scales with temperature—especially during wet days—remains relatively underexplored^28–31^, even though this information is crucial for improving our understanding of the moisture levels that effectively contribute to the development of extreme precipitation events. It’s worth noting that water vapor refers to the moisture content of the atmosphere, which can be measured in different ways, such as specific humidity, relative humidity, saturation vapor pressure, and dew point temperature. Only a few studies^27–32^ have examined the scaling of water vapor with temperature at different pressure levels and attributed to the challenges in obtaining accurate and consistent humidity data at different altitudes^33–36^. Relative humidity, in particular, is highly sensitive to temperature errors and is often less reliable in reanalysis products compared to specific humidity^37,38^. In this study, we focused on specific humidity over regions of Japan, where only a few studies have investigated its relationship with temperature during wet days, and those have been limited to either the surface level^7^ or column-integrated water vapor^29^. Furthermore, previous studies have mostly focused on exploring the existence of a CC-like scaling for extreme precipitation events at various temporal and spatial scales in present and future climates^19,20,39−41^. Although these studies have revealed the heterogeneity of the relationship depending on various factors such as precipitation pattern, topography, and location, it remains unclear whether the CC-like scaling exists in all regions of Japan^14,42^. To address this knowledge gap, our study investigated the regional variation of the CC-like scaling of extreme precipitation events across the coast, interior, and seven regions of Japan (Fig. 1a) in the present climate (1951—2010) and the potential changes in the scaling over the next 100 years (2051—2110). We then examined the scaling of moisture content at different pressure levels of the troposphere with respect to temperature during wet days in the same seven regions of Japan. Specifically, we explored the vertical profile of the rate of change of specific humidity with temperature on wet days to provide novel insights into the dynamics of moisture and precipitation extremes over Japan regions. To the best of our knowledge, this study is the first to investigate the scaling of water vapor at multiple pressure levels of the free troposphere and its direct relationship to temperature, particularly in the context of extreme precipitation events over Japan in present climate and future warming conditions.

## Results

The study produced two main results. Firstly, the study examined CC-like relationships over the coast, interior and seven regions of Japan using the Asian Precipitation—Highly—Resolved Observational Data Integration Towards Evaluation (APHRODITE^43^ daily data and the database for Policy Decision making for Future climate change (d4PDF^44^ dataset. The datasets were available for the periods 1951—2010 (50 ensemble experiment results) and 2051—2110 (90 ensemble experiment results). Secondly, the study explored the scaling of water vapor at different tropospheric pressure levels using one ensemble experiment for the present climate and one ensemble experiment for the future climate and Modern-Era Retrospective analysis for Research and Applications, Version 2 (MERRA-2^45^) data for the period (1980–2022).

### Regional variation of the scaling over Japan

The probability distribution functions (PDFs) of precipitation amounts computed from the APHRODITE daily observation on wet days in seven regions of Japan are shown in Fig. 1b and the surface air temperatures on the corresponding wet days observed by APHRODITE are shown in Fig. 1c. Here, a wet day is defined as a day on which the regional averaged daily precipitation amount exceeds 1 mm. The highest precipitation amount is found in the Okinawa region, with daily precipitation amounts of 266 mm. In contrast, the northern regions experience relatively weaker wet events, with daily precipitation amounts below 80 mm. The PDFs of the temperature on the wet days indicate a variation primarily depending on the latitudinal zones and local climates. Wet days over the Okinawa region have relatively more frequent warmer days with temperatures around 23 °C, followed by those over the western regions and Pacific Ocean side of eastern regions (15–17 °C), and northern regions (6–10 °C). These findings suggest that the precipitation patterns and corresponding temperature shapes are not uniform across the regions of Japan, and thus, the relationship between the two over the seven regions is expected to differ.

**Fig. 2:**
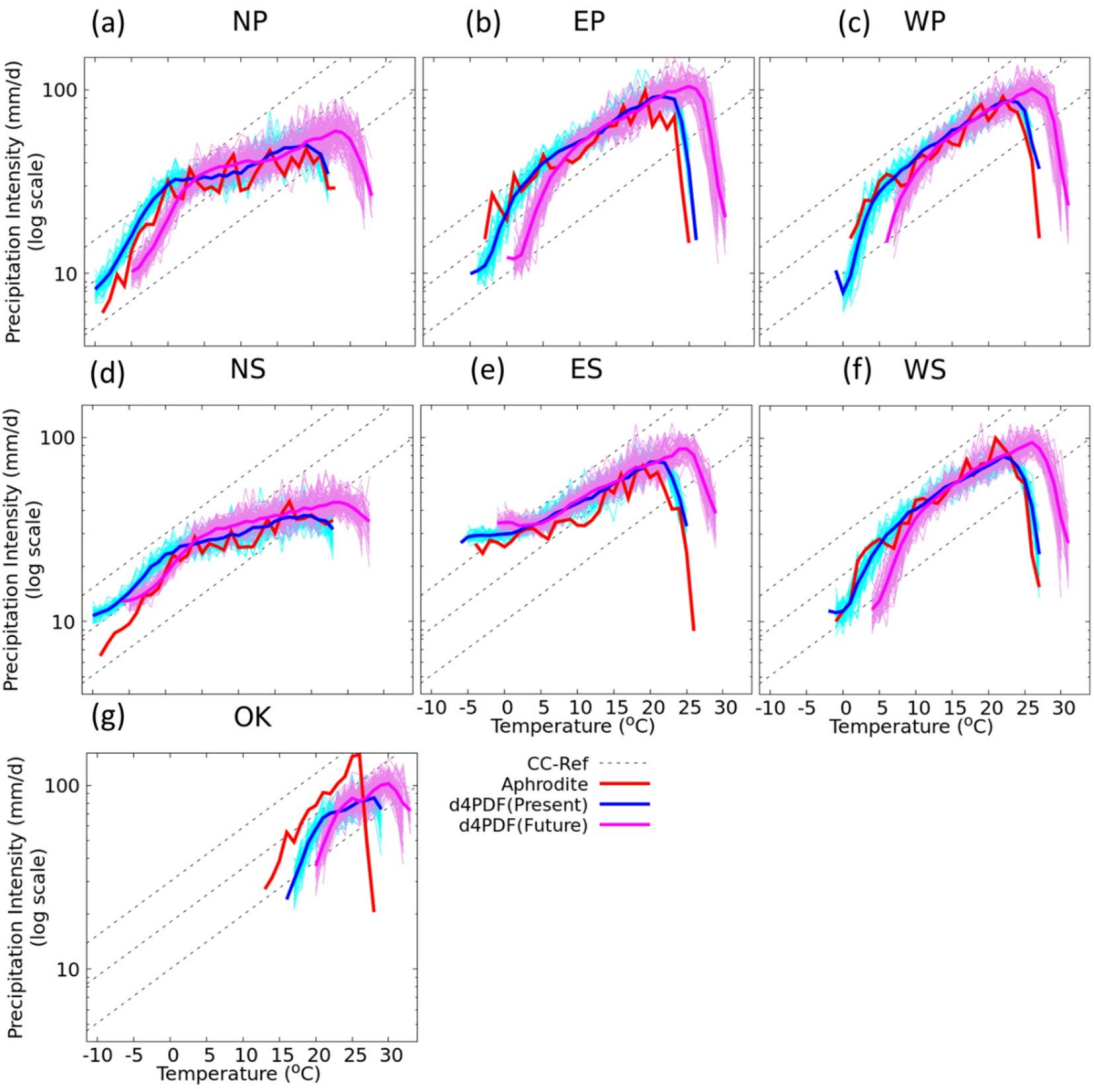
Scaling of 99th percentile daily precipitation with temperature on the wet days computed from APHRODITE data and d4PDF datasets over North Japan (**a**, **d**), East Japan (**b**, **e**), West Japan (**c**, **f**) and Okinawa (**g**). The CC relationship is used as a reference. Thin lines show the relationship from each individual ensemble members (50 ensemble members for present climate and 90 ensemble members for future climate). The thick red line represents the scaling from APHRODITE data, and thick blue and magenta represent the scaling of the mean from the present and future ensemble members respectively.

The 99th percentile precipitation events linked to temperature exhibit a CC-like relationship in all seven regions up to a certain temperature, which is consistent with previous studies conducted over Japan^14,15,19^. The characteristics of the relationship found to be qualitatively similar among the regions of Japan, but they differ quantitatively. The CC-like relationship of precipitation with temperature is found to exist up to 21 °C in the present climate ensemble over the northern regions (Fig. 2a and d), while it is observed up to 27 °C over the Okinawa region (Fig. 2g). Such a CC-like relationship is observed up to 22–23 °C in the other regions (Fig. 2b,c,e,f). These results closely follow the relationships found in the APHRODITE data over all the regions except Okinawa. In Okinawa, the d4PDF simulations underestimate the magnitude of 99th percentile precipitation by approximately 25–46% with the 1 °C temperature bins compared to APHRODITE observations (Table S1). One of the reasons for the underestimates of those extreme values in Okinawa is because the d4PDF (having a spatial resolution of 20 km) is not able to represent properly the land-sea contrast and topography of the Okinawa islands. Despite this bias in absolute values, the CC-like scaling pattern—showing precipitation increasing with temperature up to a peak and then declining—is preserved. The relationships between extreme precipitation events and temperature in the future warming climate over all regions are expected to extend to a higher temperature range by another 2–4 °C. Therefore, the intensities of extreme precipitation events in the future climate are also anticipated to increase, by about 10 mm/d over NP, EP, and NS (Fig. 2a,b,d) and by ~ 20 mm/d over the other regions (Fig. 2c and e-g). We tested the sensitivity of our results to the definition of a wet day by applying different thresholds of 0.5, 2, 5, and 10 mm. The scaling pattern remained consistent with all thresholds. The precipitation intensities also showed a trend increasing with temperature up to a peak and then declining thereafter with all thresholds (Fig. S1). The higher thresholds shifted the 99th percentile values to higher magnitudes, which is obvious since the percentiles reflect stronger precipitation events. However, the overall CC-like scaling behavior remained unchanged.

**Fig. 3:**
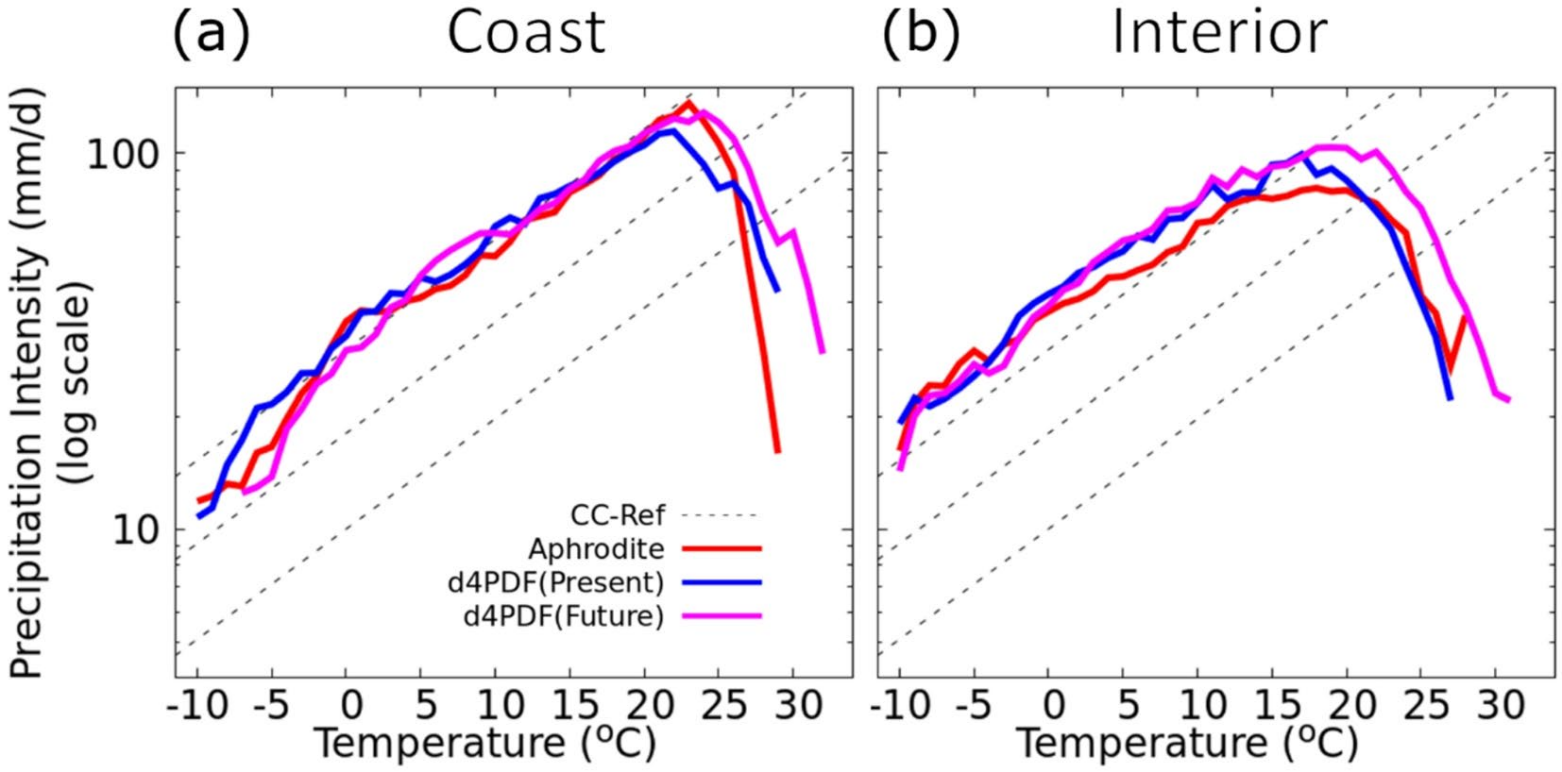
Scaling of 99th percentile daily precipitation with temperature on the wet days computed from APHRODITE data and d4PDF datasets over the (**a**) coast (left) and the (**b**) interior of Japan. The thick red line represents the scaling from APHRODITE data, and thick blue and magenta represent the scaling of the first ensemble member from the present and future d4PDF dataset.

We further extended the analysis to the Japan interior and coast regions (randomly selected 30 interior grid points located at least 50 km away from the coast, and 60 points along the Japan coast as shown in Fig. 1). We found that both interior and coast of Japan exhibit a CC-like scaling relationship with temperature up to a certain threshold, similar to the patterns observed across the seven regions of Japan (Fig. 3). Interestingly, the coast exhibited this CC-like relationship up to approximately 23 °C, whereas the interior showed only up to around 20 °C. This extended range in the coast may be attributed to the moderating influence of the ocean, which sustains higher humidity levels and supports more intense precipitation at higher temperatures compared to the interior. In the future warming climate, both regions exhibit an increase in the intensities of extreme precipitation events, consistent with the trends observed in other parts of Japan.

### Vertical variation of the scaling of the water vapor

We conducted an analysis on the scaling of water vapor with respect to temperature at 14 different pressure levels on wet days across seven regions of Japan.

**Fig. 4:**
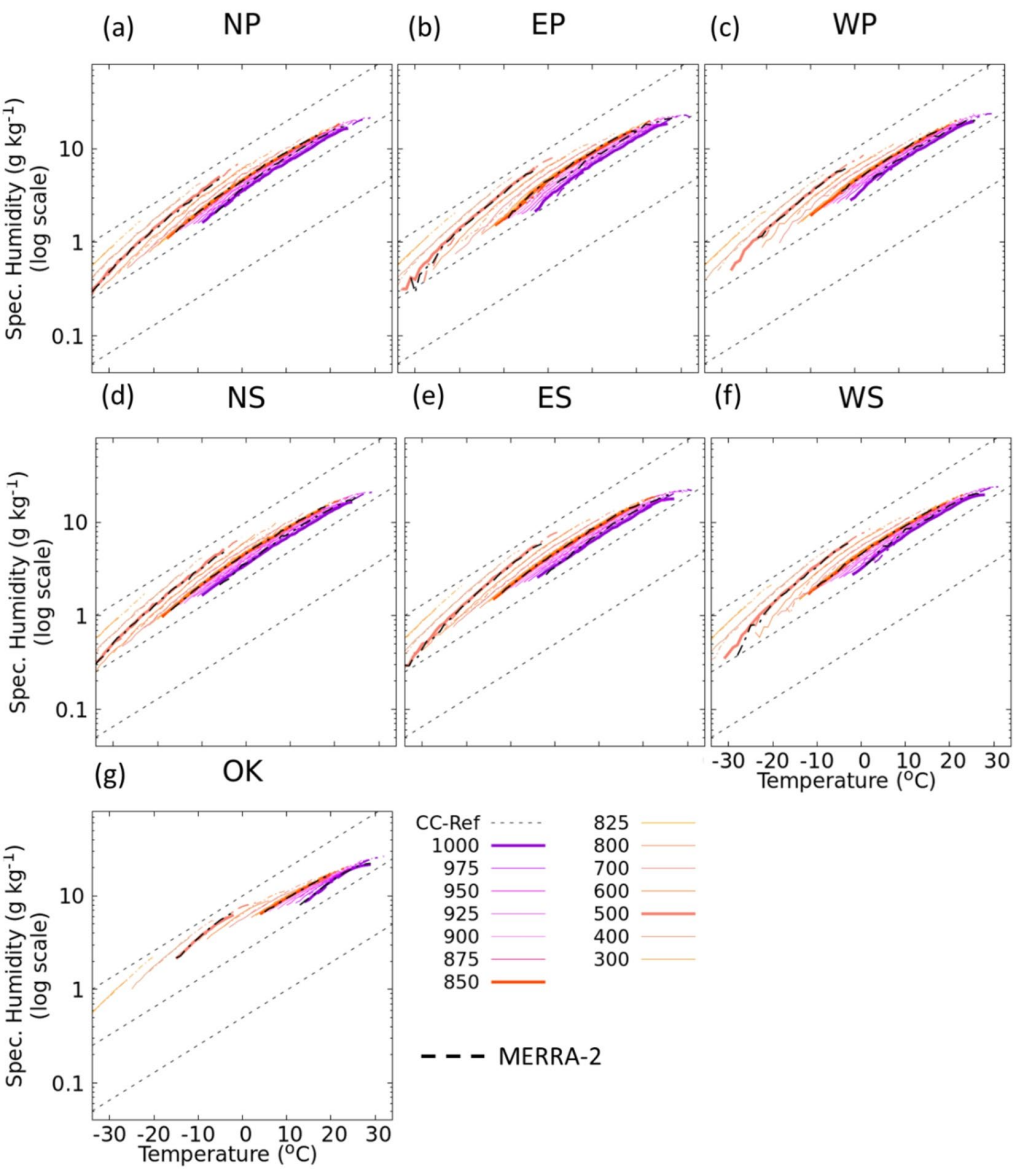
The 99th percentile specific humidity as a function of temperature during the wet events at 14 pressure levels (1000 hPa―300 hPa) over North Japan (**a**, **d**), East Japan (**b**, **e**), West Japan (**c**, **f**) and Okinawa (**g**). Solid (Dashed) lines correspond to the results in present (future). Thicker violet, deep-orange and orange lines indicate the results at 1000, 850 and 500 hPa respectively. The dashed thick black line represents the results from the MERRA-2 data at 1000, 850 and 500 hPa.

Our results indicate that the relationship between specific humidity and temperature follows a CC-like relationship at lower pressure levels, but not as clearly at higher levels (Fig. 4). While the qualitative patterns of this relationship appear similar across all regions of Japan, the specific humidity value itself is higher in southern regions and lower in northern regions. For clarity, we focus on the results found at the 1000, 850, and 500 hPa levels. The 99th percentile specific humidity on wet days at these levels increases with temperature across all regions of Japan, with a slight decrease at higher temperatures. This scaling of specific humidity holds up to around 23 °C, which is consistent with extreme precipitation events (Fig. 2b,c,f). However, the scaling of specific humidity with temperature found to be weaker above 500 hPa, perhaps due to the limited amount of water vapor available at this level or no significant relationship between moisture at this level and extreme precipitation events^46–48^. To assess the robustness of the specific humidity data used in our study, we compared our results with those derived from the MERRA-2 reanalysis dataset. The comparison at the three focused levels (1000, 850, and 500 hPa) showed consistent scaling behavior across the regions of Japan, indicating robust trends obtained from the d4PDF datasets. Our analysis of the scaling of specific humidity with temperature in the future warming climate shows similar characteristics as that in the present climate, but the relationship extends to higher temperature values by another 4 °C. This upward shift reflects the warming-induced increase in atmospheric moisture holding capacity, especially in the lower to mid-troposphere, where strong correlations with precipitation extremes are found. The results are supported by global studies that also identify this vertical moisture structure, particularly between 850 and 400 hPa as a key driver of precipitation intensification^31^ and suggests that the moisture content available for precipitation would increase by about 5 g/kg in the future warming climate, implying more enhanced precipitation events^25,49–51^. A closer examination of the specific humidity scaling shown in Fig. 4 reveals small deviations from the CC reference at all pressure levels. We consider that these small deviations could be used to generate stronger precipitation events.

The scaling of relative humidity with temperature on wet days is not observed on any levels up to 300 hPa (Fig. S2). The relative humidity values at 850 hPa are found to be the highest among all the pressure levels at various temperature values in all regions of Japan. The higher relative humidity values at lower (below 850 hPa) and middle levels (850 − 500 hPa) indicate favorable conditions for ascending air parcels to develop thunderstorms^52–57^. Notably, relative humidity values at all levels below 300 hPa show a decreasing trend above a certain temperature across all regions. This decreasing trend may play a role in weakening the precipitation intensity after a certain temperature^12^, as observed earlier (Fig. 2). The relative humidity in the future climate does not show any changes from the present climate, but maintains the same trend up to another 4 °C. This may have a significant impact on precipitation production, particularly when relative humidity values are maintained above 80%. To further understand this relationship, we analyzed the relationship between relative humidity and precipitation events, as shown in Fig. S3. Our findings indicate that extreme precipitation event intensity increases with relative humidity, particularly at lower levels (below 850 hPa), and stronger precipitation events with intensity above the 99th percentile are associated with higher relative humidity (mostly above 80%). These results suggest that maintaining higher relative humidity values can lead to stronger precipitation events.

**Fig. 5:**
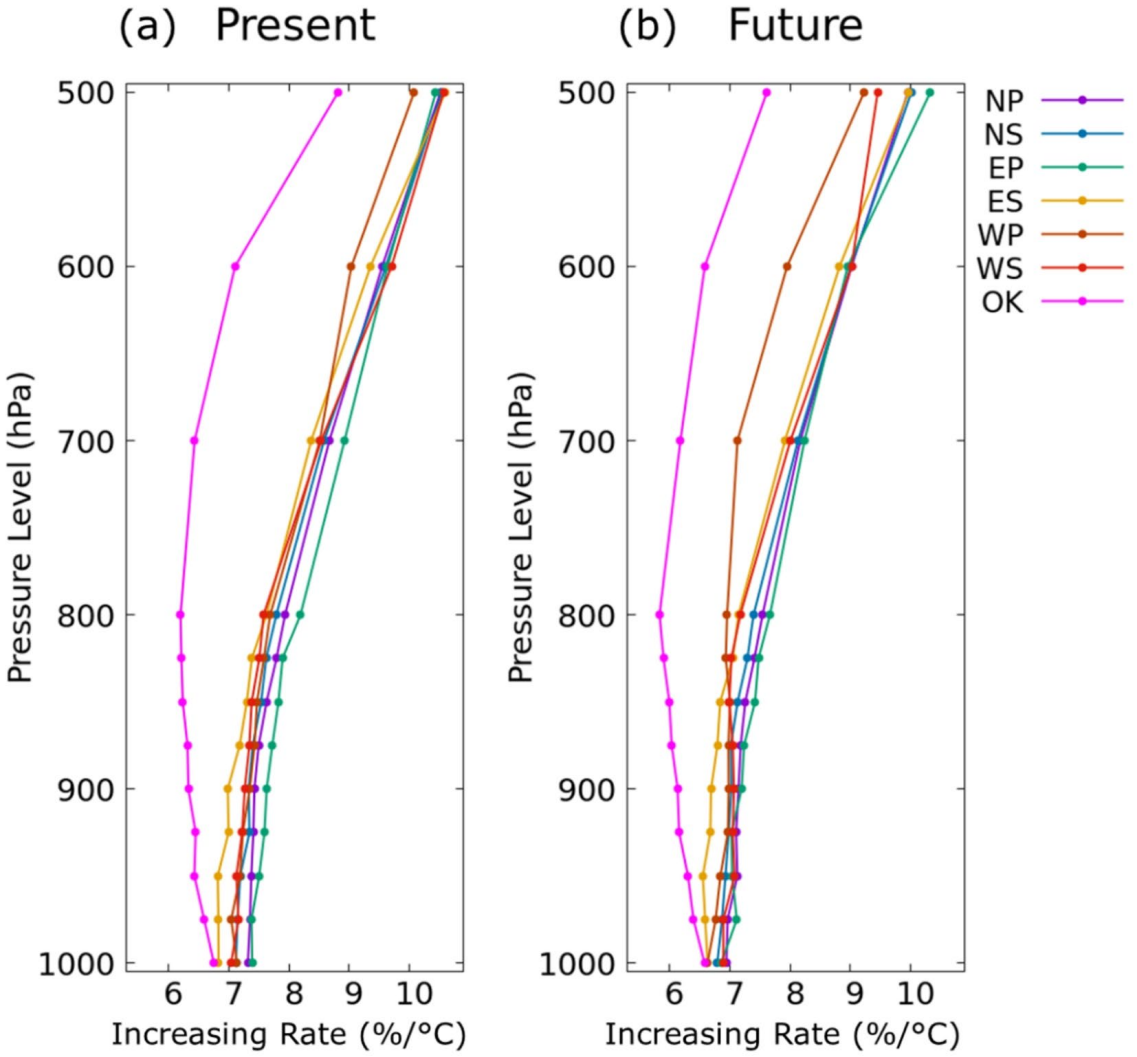
The rate of change of 99th percentile specific humidity for the levels 1000 hPa through 500 hPa with respect to temperature in (**a**) present climate and (**b**) future climate.

The rate of change of the 99th percentile specific humidity with respect to temperature between the layers from 1000 hPa through 500 hPa on the wet days is shown in Fig. 5. The rate over OK decreases with altitude up to 800 hPa and then increases, while the rate over ES shows an increase with altitude. The rates over other regions either show no change or a small increase up to 850 hPa, followed by an increase above the 850 hPa level. The rate is higher at upper levels than at lower levels, and it varies with atmospheric levels within 8.3 ± 2.4%/°C in each region. At the lowest level (1000 hPa), the rate is 6.8–7.3%/°C, which closely follows the CC-scaling. A closer investigation reveals that the rate is higher over northern regions and lower over southern regions, indicating higher rates at cooler temperatures and lower rates at warmer temperatures. The rates of change of specific humidity in the future climate do not show significant changes. The profiles of the rates of change of specific humidity over OK, NP, NS, and ES remain mostly the same as in the present climate, while those over EP, WP, and WS slightly decrease with altitude above the 900 hPa. However, the rates between the two climate periods are not significantly different. Particularly at 850 hPa, where the relative humidity shows maximum values, the rates follow closely the CC-scaling in all the regions except OK. The rate over OK follows closely the CC-scaling at the 1000 hPa level, while the rates over other regions follow the CC-scaling up to 850 hPa.

## Discussion

The intensity of precipitation events is expected to increase in the future climate across all seven regions of Japan. Previous studies have discussed the relationship between precipitation and temperature in terms of moisture availability^12,28,58^. Our analysis shows that specific humidity is increasing at all pressure levels (Fig. 4), which will contribute to generating extreme precipitation events in the future climate. Additionally, relative humidity at 850 hPa is projected to remain above 80% over a wider temperature range (Fig. S2). Among all regions, precipitation intensity is strongest in OK, and is expected to increase even more under a warmer climate due to higher specific humidity in this region, making it more vulnerable to extreme precipitation events. To illustrate the broader relevance of our findings, we compare them with studies from other regions highlighting the role of tropospheric moisture in extreme precipitation. Holloway and Neelin^48^ showed that free-tropospheric moisture (850–200 hPa) strongly controls precipitation intensification. Bretherton et al.^31^ found an exponential relationship between water vapor path and precipitation, particularly influenced by humidity between 400 and 850 hPa. Roderick et al.^30^ reported contrasting scaling at two Australian sites (− 44.1%/°C at Darwin vs. +11.7%/°C at Mildura), emphasizing regional variability. Similarly, Bui et al.^27^ found increases of ~ 7%/°C in Australian cities, consistent with our rate of 8.3 ± 2.4%/°C across Japan. Ali and Mishra^26^ further observed super-CC scaling in Indian urban areas. These studies confirm that atmospheric moisture strongly governs extreme precipitation globally and support the robustness of our findings in the context of a warming climate.

Our further analysis reveals that the intensities of extreme precipitation events will continue to increase with a temperature range that is 2–4 °C higher in the future climate than in the present climate across all regions of Japan. To understand the mechanism behind this intensification, we analyzed the temperature lapse rate between 850 and 500 hPa on wet days (Fig. S4). We found that the lapse rates in the lower temperature range remain almost unchanged between the present and future climates. There is a slight decrease in the lapse rate with increasing temperature and the difference between the present and future climates is negligible. This suggests that the atmospheric stability in terms of temperature lapse rate on wet days remains the same between the present climate and the warming scenario. To further investigate the physical drivers of intensification, we analyzed vertical velocity (omega) at 700 hPa during extreme precipitation events (Fig. S5). The results show a notable increase in upward motion in the future climate, suggesting enhanced convective activity. This stronger ascent facilitates the vertical transport of moisture and contribute to more intense precipitation events. When considered alongside the extended scaling of specific humidity with temperature, our findings indicate that while the thermodynamic contribution (via increased moisture availability) remains dominant, dynamic factors such as increased vertical motion further amplify extreme precipitation under future warming scenarios. The moisture contents on wet days are likely to increase in the future warming climate (Fig. 4), which, combined with an unchanged temperature lapse rate, but thermodynamic contribution, should play a role in enhancing the increased extreme precipitation events^11,59^. Although increased moisture availability sets the overall scaling potential, circulation processes such as enhanced low-level convergence, changes in storm tracks, or shifts in the East Asian summer monsoon may act to either strengthen or offset this thermodynamic signal. This interaction likely explains part of the regional variability seen in our results, for instance the weaker scaling in northern regions compared to the south. Although a detailed analysis of circulation drivers was beyond the scope of this study, our findings highlight the importance of considering both thermodynamic and dynamic contributions when interpreting precipitation–temperature scaling under climate change. Pfahl et al.^32^ highlighted that the dynamic contribution of atmospheric circulation to extreme precipitation changes can either amplify or weaken the thermodynamic response, depending on the region. Furthermore, over subtropical oceans, shifts in circulation patterns can even cause robust regional decreases in extreme precipitation, likely due to a poleward shift in circulation.

Our results indicate that while specific humidity generally follows CC scaling across all regions (Fig. 4), extreme precipitation (Fig. 2) does not always exhibit the same rate of increase with temperature, especially in cooler northern regions (Fig. 2a and d). This discrepancy suggests that factors beyond moisture availability, such as changes in relative humidity may influence the efficiency of precipitation formation. Indeed, Fig. S2 shows a decrease in relative humidity at higher temperatures, which could suppress convective activity or alter the vertical moisture distribution, thus limiting precipitation intensities even when specific humidity continues to rise. However, this relationship is not always universal and may vary depending on regional and dynamic factors. Our study primarily focused on the thermodynamic contributions of increased moisture in the atmosphere due to warming, which generally leads to more intense precipitation. Therefore, future research is suggested to explore how changes in atmospheric circulation, including shifts in storm tracks, jet streams, and monsoon patterns, interact with thermodynamic changes to affect regional extreme precipitation events over Japan. It should also be noted that the analysis of vertical humidity scaling in this study was performed using one representative ensemble member for the present climate and one for the future climate. The humidity–temperature relationships may vary across ensemble members due to internal variability, and therefore we confirmed the robustness of our results by comparing them with independent MERRA-2 reanalysis data, which showed very close consistency across multiple levels and regions (Fig. 4). Thus, we consider that the analysis based on the single ensemble member demonstrates a robust signal in the humidity-temperature relationship. However, future research could use multiple members to quantify ensemble spread and better capture the uncertainty range of scaling rates.

## Methods

We analyzed the Policy Decision making for Future climate change (d4PDF) dataset available hourly at a 20 km resolution for the periods 1951—2010 (50 ensemble experiment results) and 2051—2110 (90 ensemble experiment results) to investigate the impact of future climate change on precipitation in Japan. These ensemble simulations were conducted for the historical 3000 years and the future 5400 years with various initial conditions and small perturbations of sea surface temperatures^44^. Specifically, the historical (future) 3000 (5400) years refer to 50 (90) ensemble experiments, each spanning 60 years, adding up to 50 (90) x 60 years. We used the Asian Precipitation—Highly—Resolved Observational Data Integration Towards Evaluation (APHRODITE^43^ daily datasets at 0.25 degrees for the period 1961—2015 as observational data for validating the d4PDF precipitation analysis. The analysis procedure involves stratifying wet days (defined as daily precipitation amount greater than 1 mm) into 1 °C temperature bins and computing the 99th percentile in each temperature bin over each of the seven regions of Japan, as shown in Fig. 1a. These regions were classified based on previous studies^60,61^. The specific humidity and relative humidity were analyzed from one ensemble experiment each, namely the first experiment (HPB_m001) for the present climate, and HFB_4K_MI_m101 for the future climate, which utilized the warming data from the Model for Interdisciplinary Research on Climate (MIROC5). The Data Integration and Analysis System (DIAS) (http://search.diasjp.net/en/dataset/d4PDF_GCM) contains detailed information about these two ensemble members (HPB_m001 and HFB_4K_MI_m101). Similar to the method used for analyzing the relationship between precipitation and temperature, the specific humidity and relative humidity on wet days were stratified into 1 °C temperature bins and analyzed separately for 14 atmospheric levels. The rate of change of specific humidity with temperature at different pressure levels was calculated using the following equation:

1 $$P_{2} = P_{1} (1 + \alpha )^{{\Delta T}}$$

Where *P*_*1*_ and *P*_*2*_ are the specific humidities on two precipitation events, *T*_*1*_ and *T*_*2*_ are the temperatures of the two corresponding precipitation events; *∆T = T*_*2*_*—T*_*1*_, and *α* is the rate of change of the specific humidity during the precipitation events. Here, α is approximately equal to 0.07, which corresponds to the CC-scaling relationship that has been previously established^6,7,29^. Modern-Era Retrospective analysis for Research and Applications, Version 2 (MERRA-2^45^), data for the period 1980–2022 was used to validate the specific humidity scaling with temperature obtained from the d4PDF datasets. MERRA-2 is a NASA reanalysis dataset (https://gmao.gsfc.nasa.gov/reanalysis/merra-2/) produced by the Global Modeling and Assimilation Office (GMAO), designed to provide a consistent, long-term record of atmospheric data from 1980 onward.

## Supplementary Information

Below is the link to the electronic supplementary material.

Supplementary Material 1

## Data Availability

This study utilized the database for Policy Decision-making for Future climate change (d4PDF) which is available at [http://search.diasjp.net/en/dataset/d4PDF\_RCM] , the Asian Precipitation-Highly-Resolved Observational Data Integration Towards Evaluation (APHRODITE) daily datasets which is available at [https://www.chikyu.ac.jp/precip/english] , and the Modern-Era Retrospective analysis for Research and Applications, Version 2 (MERRA-2) data which is available at [https://gmao.gsfc.nasa.gov/reanalysis/merra-2](https:/gmao.gsfc.nasa.gov/reanalysis/merra-2) .

## References

[1] Intergovernmental Panel on Climate Change (IPCC). *Managing the Risks of Extreme Events and Disasters To Advance Climate Change adaptation. A Special Report of Working Groups I and II of the Intergovernmental Panel on Climate Change [Field]*. 582 (eds Barros, C. B. V., Stocker, T. F., Qin, D., Dokken, D. J., Ebi, K. L., Mastrandrea, M. D., Mach, K. J., Plattner, G. K., Allen, S. K., Tignor, M. & Midgley, P. M.) (Cambridge University Press, 2012).

[2] Intergovernmental Panel on Climate Change (IPCC). : Impacts, Adaptation, and Vulnerability. Part A: Global and Sectoral Aspects. Contribution of Working Group II to the Fifth Assessment Report of the Intergovernmental Panel on Climate Change (Eds C.B. Field, V.R. Barros, D.J. Dokken, K.J. Mach, M.D. Mastrandrea & T.E. Bilir et al.), 1132. (Cambridge University Press, 2014).

[3] Mori, N. et al. Recent nationwide climate change impact assessments of natural hazards in Japan and East Asia. *Weather Clim. Extremes*. **32**, 100309 (2021).

[4] Raymond, C. et al. Understanding and managing connected extreme events. *Nat. Clim. Change*. **10** (7), 611–621 (2020).

[5] Bao, J., Sherwood, S. C., Alexander, L. V. & Evans, J. P. Future increases in extreme precipitation exceed observed scaling rates. *Nat. Clim. Change*. **7** (2), 128–132 (2017).

[6] Nayak, S., Dairaku, K., Takayabu, I. & Suzuki-Parker, A. Ishizaki, N. N. Extreme precipitation linked to temperature over japan: current evaluation and projected changes with multi-model ensemble downscaling. *Clim. Dyn.* **51** (11), 4385–4401 (2018).

[7] Nayak, S. & Takemi, T. Clausius-Clapeyron scaling of extremely heavy precipitations: case studies of the July 2017 and July 2018 heavy rainfall events over Japan. *J. Meteorol. Soc. Jpn*. **98** (6), 1147–1162 (2020).

[8] Trenberth, K., Dai, A., Rasmussen, R. & Parsons, D. The changing character of precipitation. *Bull. Am. Meteorol. Soc.* **84** (9), 1205–1217 (2003).

[9] Skliris, N., Zika, J. D., Nurser, G., Josey, S. A. & Marsh, R. Global water cycle amplifying at less than the Clausius-Clapeyron rate. *Sci. Rep.* **6** (1), 1–9 (2016). 27934946 10.1038/srep38752 PMC5146653

[10] Zhang, W., Zhou, T., Zhang, L. & Zou, L. Future intensification of the water cycle with an enhanced annual cycle over global land monsoon regions. *J. Clim.* **32** (17), 5437–5452 (2019).

[11] Westra, S. et al. Future changes to the intensity and frequency of short-duration extreme rainfall. *Rev. Geophys.* **52** (3), 522–555 (2014).

[12] Hardwick, J. R., Westra, S. & Sharma, A. Observed relationships between extreme sub-daily precipitation, surface temperature, and relative humidity. *Geophys. Res. Lett.* **37**, L22805 (2010).

[13] Lenderink, G. & Van Meijgaard, E. Increase in hourly precipitation extremes beyond expectations from temperature changes. *Nat. Geosci.* **1** (8), 511–514 (2008).

[14] Utsumi, N., Seto, S., Kanae, S., Maeda, E. E. & Oki, T. Does higher surface temperature intensify extreme precipitation? *Geophys. Res. Lett.* **38**, L16708 (2011).

[15] Nayak, S. & Dairaku, K. Future changes in extreme precipitation intensities associated with temperature under SRES A1B scenario. *Hydrol. Res. Lett.* **10** (4), 139–144 (2016).

[16] Nayak, S. Do extreme precipitation intensities linked to temperature over India follow the Clausius-Clapeyron relationship? *Curr. Sci.* **115** (3), 391–392 (2018).

[17] Moustakis, Y., Onof, C. J. & Paschalis, A. Atmospheric convection, dynamics and topography shape the scaling pattern of hourly rainfall extremes with temperature globally. *Commun. Earth Environ.* **1** (1), 1–9 (2020).

[18] Drobinski, P. et al. Scaling precipitation extremes with temperature in the mediterranean: past climate assessment and projection in anthropogenic scenarios. *Clim. Dyn.* **51** (3), 1237–1257 (2018).

[19] Fujita, M. et al. Precipitation changes in a climate with 2-K surface warming from large ensemble simulations using 60‐km global and 20‐km regional atmospheric models. *Geophys. Res. Lett.* **46** (1), 435–442 (2019).

[20] Pauluis, O. & Held, I. M. Entropy budget of an atmosphere in radiative–convective equilibrium. Part II: latent heat transport and moist processes. *J. Atmos. Sci.* **59** (2), 140–149 (2002).

[21] Zhang, Y. & Fueglistaler, S. Mechanism for increasing tropical rainfall unevenness with global warming. *Geophys. Res. Lett.* **46** (24), 14836–14843 (2019).

[22] Trenberth, K. E. Changes in precipitation with climate change. *Climate Res.* **47** (1–2), 123–138 (2011).

[23] Trenberth, K. E., Fasullo, J. T. & Kiehl, J. Earth’s global energy budget. *Bull. Am. Meteorol. Soc.* **90** (3), 311–324 (2009).

[24] Chen, B., Liu, Z., Wong, W. K. & Woo, W. C. Detecting water vapor variability during heavy precipitation events in Hong Kong using the GPS tomographic technique. *J. Atmos. Ocean. Technol.* **34** (5), 1001–1019 (2017).

[25] Pall, P., Allen, M. R. & Stone, D. A. Testing the Clausius–Clapeyron constraint on changes in extreme precipitation under CO 2 warming. *Clim. Dyn.* **28** (4), 351–363 (2007).

[26] Ali, H. & Mishra, V. Contrasting response of rainfall extremes to increase in surface air and dewpoint temperatures at urban locations in India. *Sci. Rep.* **7** (1), 1–15 (2017). 28450745 10.1038/s41598-017-01306-1 PMC5430704

[27] Bui, A., Johnson, F. & Wasko, C. The relationship of atmospheric air temperature and dew point temperature to extreme rainfall. *Environ. Res. Lett.* **14** (7), 074025 (2019).

[28] Berg, P. et al. Seasonal characteristics of the relationship between daily precipitation intensity and surface temperature. *J. Geophys. Res.* **114**, D18102 (2009).

[29] Nayak, S. & Takemi, T. Dependence of extreme precipitable water events on temperature. *Atmósfera* **32** (2), 159–165 (2019).

[30] Roderick, T. P., Wasko, C. & Sharma, A. Atmospheric moisture measurements explain increases in tropical rainfall extremes. *Geophys. Res. Lett.* **46** (3), 1375–1382 (2019).

[31] Bretherton, C. S. & Peters, M. E. Back. Relationships between water vapor path and precipitation over the tropical oceans. *J. Clim.* **17**, 1517–1528 (2004).

[32] Pfahl, S., O’Gorman, P. A. & Fischer, E. M. Understanding the regional pattern of projected future changes in extreme precipitation. *Nat. Clim. Change*. **7** (6), 423–427 (2017).

[33] Wang, J. et al. Corrections of humidity measurement errors from the Vaisala RS80 radiosonde—Application to TOGA COARE data. *J. Atmos. Ocean. Technol.* **19** (7), 981–1002 (2002).

[34] Simmons, A. J., Willett, K. M., Jones, P. D., Thorne, P. W. & Dee, D. P. Low-frequency variations in surface atmospheric humidity, temperature, and precipitation: inferences from reanalyses and monthly gridded observational data sets. *J. Geophys. Res. Atmos.* **115** (D1), (2010).

[35] Eccel, E. Estimating air humidity from temperature and precipitation measures for modelling applications. *Meteorol. Appl.* **19** (1), 118–128 (2012).

[36] Kaufmann, S. et al. Intercomparison of midlatitude tropospheric and lower-stratospheric water vapor measurements and comparison to ECMWF humidity data. *Atmos. Chem. Phys.* **18** (22), 16729–16745 (2018).

[37] Bourdin, S., Kluft, L. & Stevens, B. Dependence of climate sensitivity on the given distribution of relative humidity. *Geophys. Res. Lett.* **48** (8), e2021GL092462. (2021).

[38] Wolf, K., Bellouin, N., Boucher, O., Rohs, S. & Li, Y. Correction of ERA5 temperature and relative humidity biases by bivariate quantile mapping for contrail formation analysis. *Atmos. Chem. Phys.* **25** (1), 157–181 (2025).

[39] Fowler, H. J. et al. Anthropogenic intensification of short-duration rainfall extremes. *Nat. Rev. Earth Environ.* 1–16 (2021).

[40] Lochbihler, K., Lenderink, G. & Siebesma, A. P. The Spatial extent of rainfall events and its relation to precipitation scaling. *Geophys. Res. Lett.* **44** (16), 8629–8636 (2017).

[41] Guerreiro, S. B. et al. Detection of continental-scale intensification of hourly rainfall extremes. *Nat. Clim. Change*. **8** (9), 803–807 (2018).

[42] Zhang, X., Zwiers, F. W., Li, G., Wan, H. & Cannon, A. J. Complexity in estimating past and future extreme short-duration rainfall. *Nat. Geosci.* **10** (4), 255–259 (2017).

[43] Yatagai, A. et al. APHRODITE: Constructing a long-term daily gridded precipitation dataset for Asia based on a dense network of rain gauges. *Bull. Am. Meteorol. Soc.* **93** (9), 1401–1415 (2012).

[44] Mizuta, R. et al. Over 5,000 years of ensemble future climate simulations by 60-km global and 20-km regional atmospheric models. *Bull. Am. Meteorol. Soc.* **98** (7), 1383–1398 (2017).

[45] Gelaro, R. et al. The modern-era retrospective analysis for research and applications, version 2 (MERRA-2). *J. Clim.* **30** (14), 5419–5454 (2017). 10.1175/JCLI-D-16-0758.1 PMC6999672 32020988

[46] Schneider, T., O’Gorman, P. A. & Levine, X. J. Water vapor and the dynamics of climate changes. *Rev. Geophys.* **48**(3) (2010).

[47] Sherwood, S. C., Roca, R., Weckwerth, T. M. & Andronova, N. G. Tropospheric water vapor, convection, and climate. *Rev. Geophys.* **48**(2) (2010).

[48] Holloway, C. E. & Neelin, J. D. Moisture vertical structure, column water vapor, and tropical deep convection. *J. Atmos. Sci.* **66** (6), 1665–1683 (2009).

[49] Polade, S. D., Gershunov, A., Cayan, D. R., Dettinger, M. D. & Pierce, D. W. Precipitation in a warming world: assessing projected hydro-climate changes in California and other mediterranean climate regions. *Sci. Rep.* **7** (1), 1–10 (2017). 28883636 10.1038/s41598-017-11285-y PMC5589768

[50] Deng, Y., Gao, T., Gao, H., Yao, X. & Xie, L. Regional precipitation variability in East Asia related to climate and environmental factors during 1979–2012. *Sci. Rep.* **4** (1), 1–13 (2014). 10.1038/srep05693 PMC4102078 25033387

[51] Feng, Z. et al. More frequent intense and long-lived storms dominate the springtime trend in central US rainfall. *Nat. Commun.* **7** (1), 1–8 (2016). 10.1038/ncomms13429 PMC5114602 27834368

[52] Takemi, T., Hirayama, O. & Liu, C. Factors responsible for the vertical development of tropical oceanic cumulus convection. *Geophys. Res. Lett.* **31**, L11109 (2004).

[53] Takemi, T. Impacts of moisture profile on the evolution and organization of midlatitude squall lines under various shear conditions. *Atmos. Res.* **82**, 37–54 (2006).

[54] Takemi, T. A sensitivity of squall line intensity to environmental static stability under various shear and moisture conditions. *Atmos. Res.* **84**, 374–389 (2007).

[55] Takemi, T. Convection and precipitation under various stability and shear conditions: squall lines in tropical versus midlatitude environment. *Atmos. Res.* **142**, 111–123 (2014).

[56] Takemi, T. & Unuma, T. Environmental factors for the development of heavy rainfall in the Eastern part of Japan during typhoon hagibis (2019). *SOLA* **16**, 30–36 (2020).

[57] Takemi, T. & Unuma, T. Diagnosing environmental properties of the July 2018 heavy rainfall event in Japan. *SOLA* **15**, A–011 (2019).

[58] Prein, A. F. et al. The future intensification of hourly precipitation extremes. *Nat. Clim. Change*. **7** (1), 48–52 (2017).

[59] O’Gorman, P. A. & Schneider, T. The physical basis for increases in precipitation extremes in simulations of 21st-century climate change. *Proc. Natl. Acad. Sci.* **106** (35), 14773–14777 (2009). 19706430 10.1073/pnas.0907610106 PMC2736420

[60] Tsunematsu, N., Dairaku, K. & Hirano, J. Future changes in summertime precipitation amounts associated with topography in the Japanese Islands. *J. Geophys. Research: Atmos.* **118**, 4142–4153 (2013).

[61] Iizumi, T., Nishimori, M., Dairaku, K., Adachi, S. A. & Yokozawa, M. Evaluation and intercomparison of downscaled daily precipitation indices over Japan in present-day climate: strengths and weaknesses of dynamical and bias correction‐type statistical downscaling methods. *J. Geophys. Res. Atmos.* **116** (D1), (2011).

