# Supplementary material for: Regional and vertical scaling of water vapor with temperature over Japan during extreme precipitation in a changing climate
Source: Sci Rep. 2025 Oct 15;15:34826. doi: 10.1038/s41598-025-22287-6 (PMC12528749; doi:10.1038/s41598-025-22287-6)
Supplement: Supplementary file 1 — Supplementary Material 1 [file 41598_2025_22287_MOESM1_ESM.docx]

Regional and Vertical Scaling of Water Vapor with Temperature over Japan during Extreme Precipitation in a Changing Climate

Sridhara Nayak*^,1^ and Tetsuya Takemi^2^

Research and Development Center, Japan Meteorological Corporation, Osaka 530-0011, Japan

Disaster Prevention Research Institute, Kyoto University, Kyoto 611-0011, Japan

**Email:**  nayak.sridhara@n-kishou.co.jp

**Supplementary figures**

**
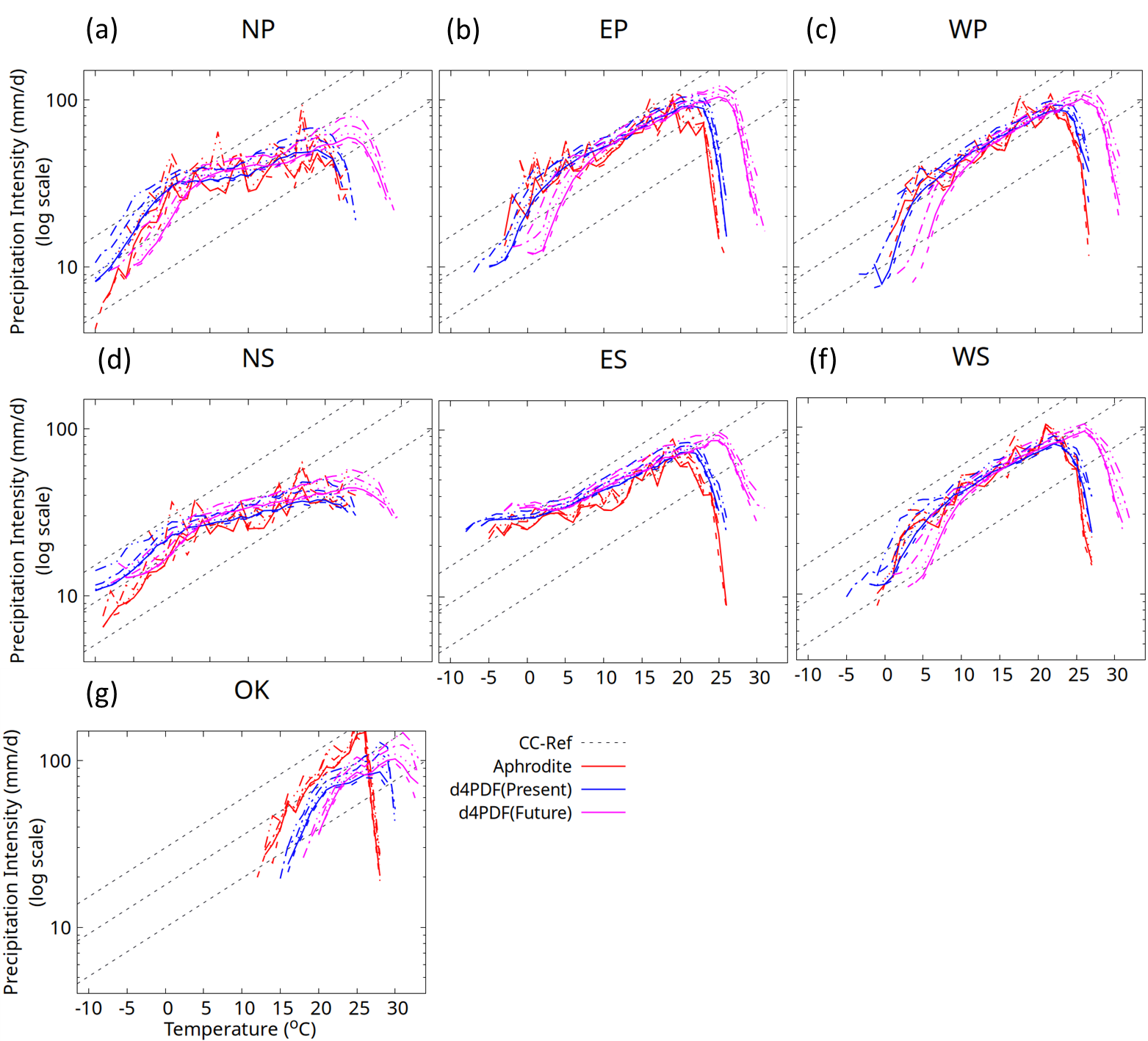
**

**Figure S1**. Sensitivity of precipitation–temperature scaling to the definition of wet days using thresholds of 0.5, 1, 2, 5, and 10 mm. The red color line represents APHRODITE and blue (magenta) color line represents the results from present (future) climate over North Japan (a, d), East Japan (b, e), West Japan (c, f) and Okinawa (g). Solid lines correspond to the results with the threshold of 1 mm and dashed lines represent the results with other thresholds.

**
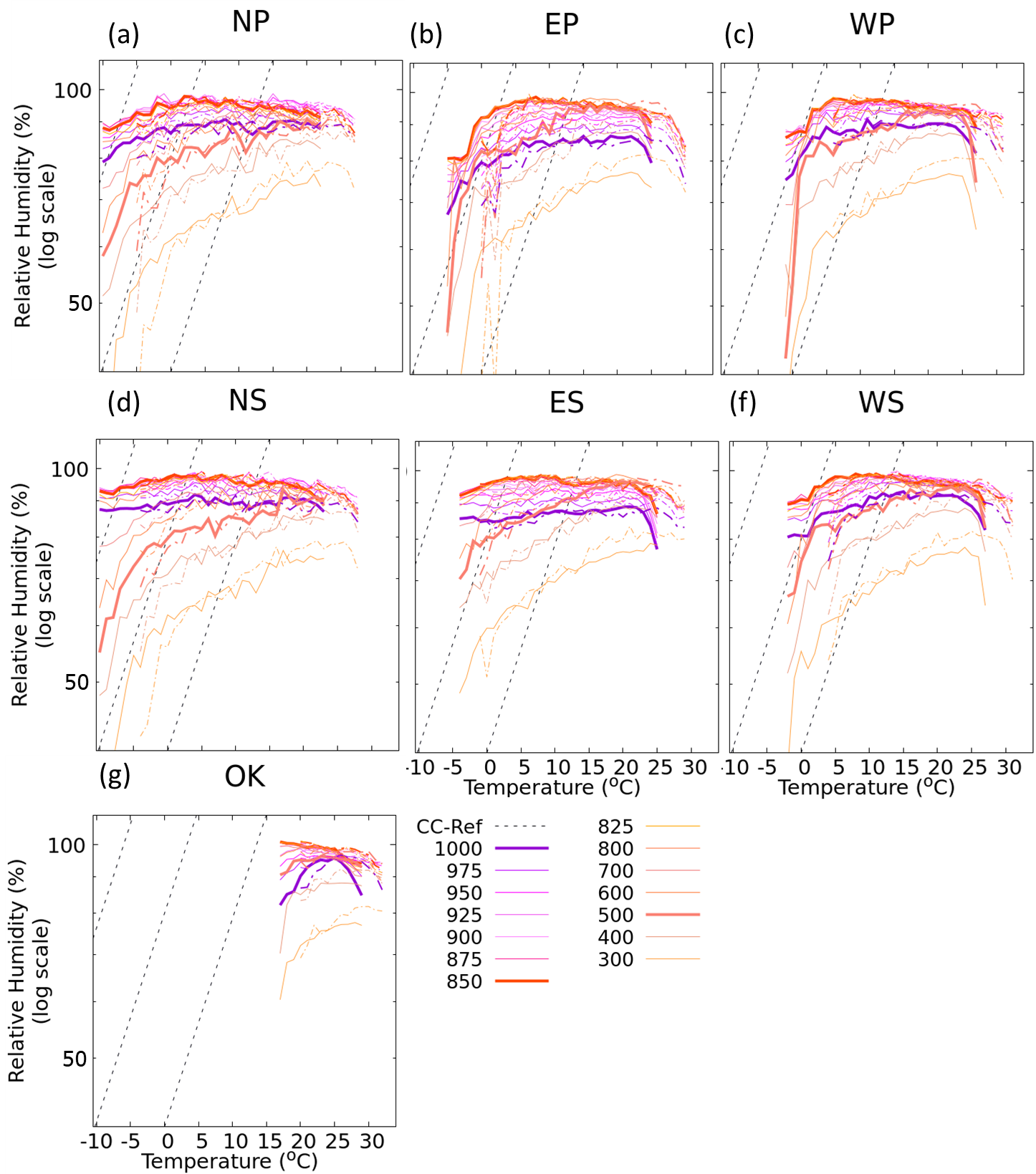
**

**Figure S2.** The 99^th^ percentile relative humidity as a function of temperature during the wet events at 14 pressure levels (1000 hPa―300 hPa) over North Japan (a, d), East Japan (b, e), West Japan (c, f) and Okinawa (g). Solid (Dashed) lines correspond to the results in present (future). Thicker violet, deep-orange and orange lines indicate the results at 1000, 850 and 500 hPa respectively.

**
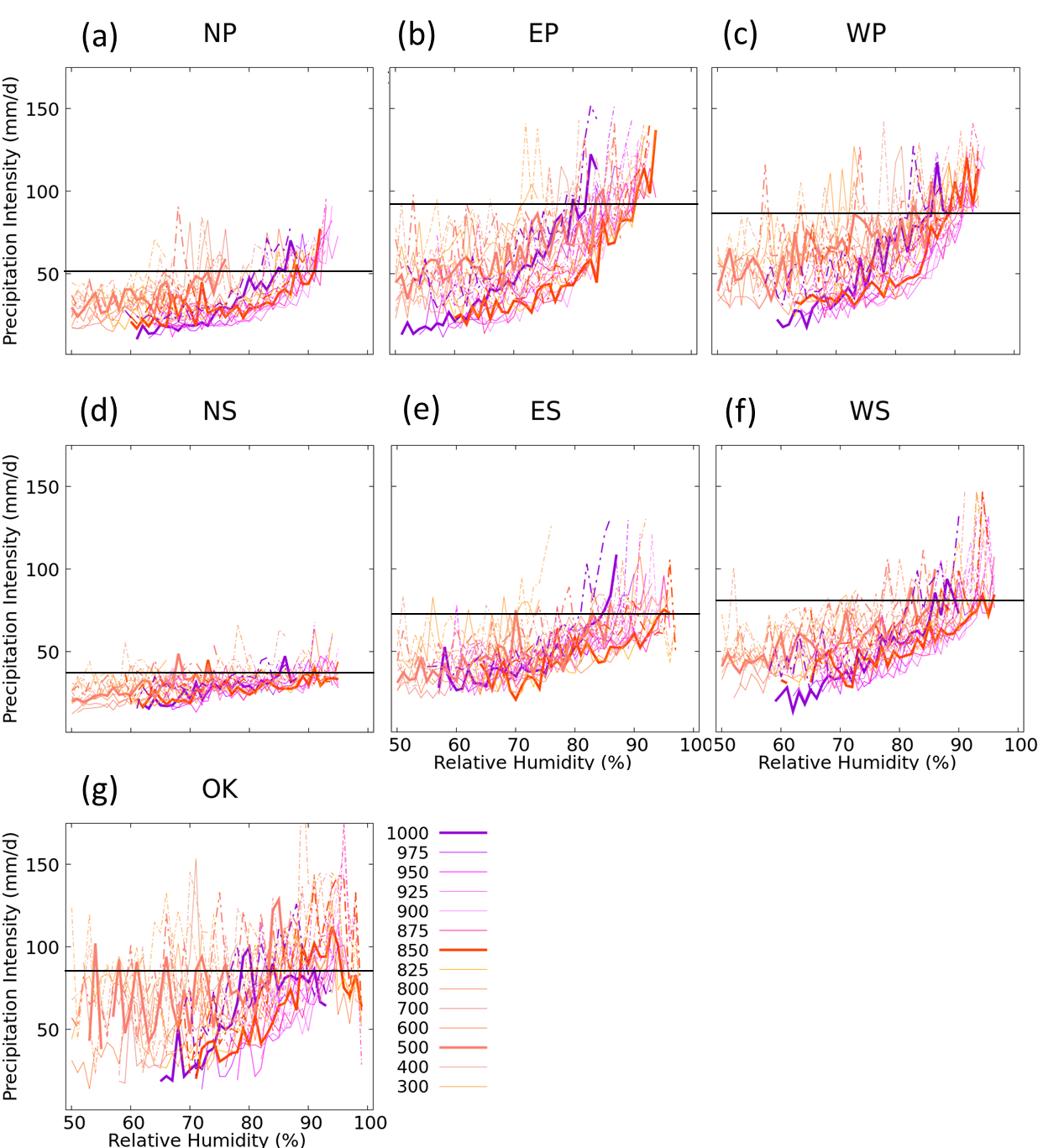
**

**Figure S3.** The 99^th^ percentile precipitation intensity as a function of relative humidity above 50% during the wet events at different atmospheric pressure levels from 1000 hPa through 300 hPa over North Japan (a, d), East Japan (b, e), West Japan (c, f) and Okinawa (g). Solid (Dashed) lines correspond to the results in present (future). Thicker violet, deep-orange and orange lines indicate the results at 1000, 850 and 500 hPa respectively. The black line corresponds to the 99^th^ percentile precipitation intensity from the ensemble mean in present climate.

**
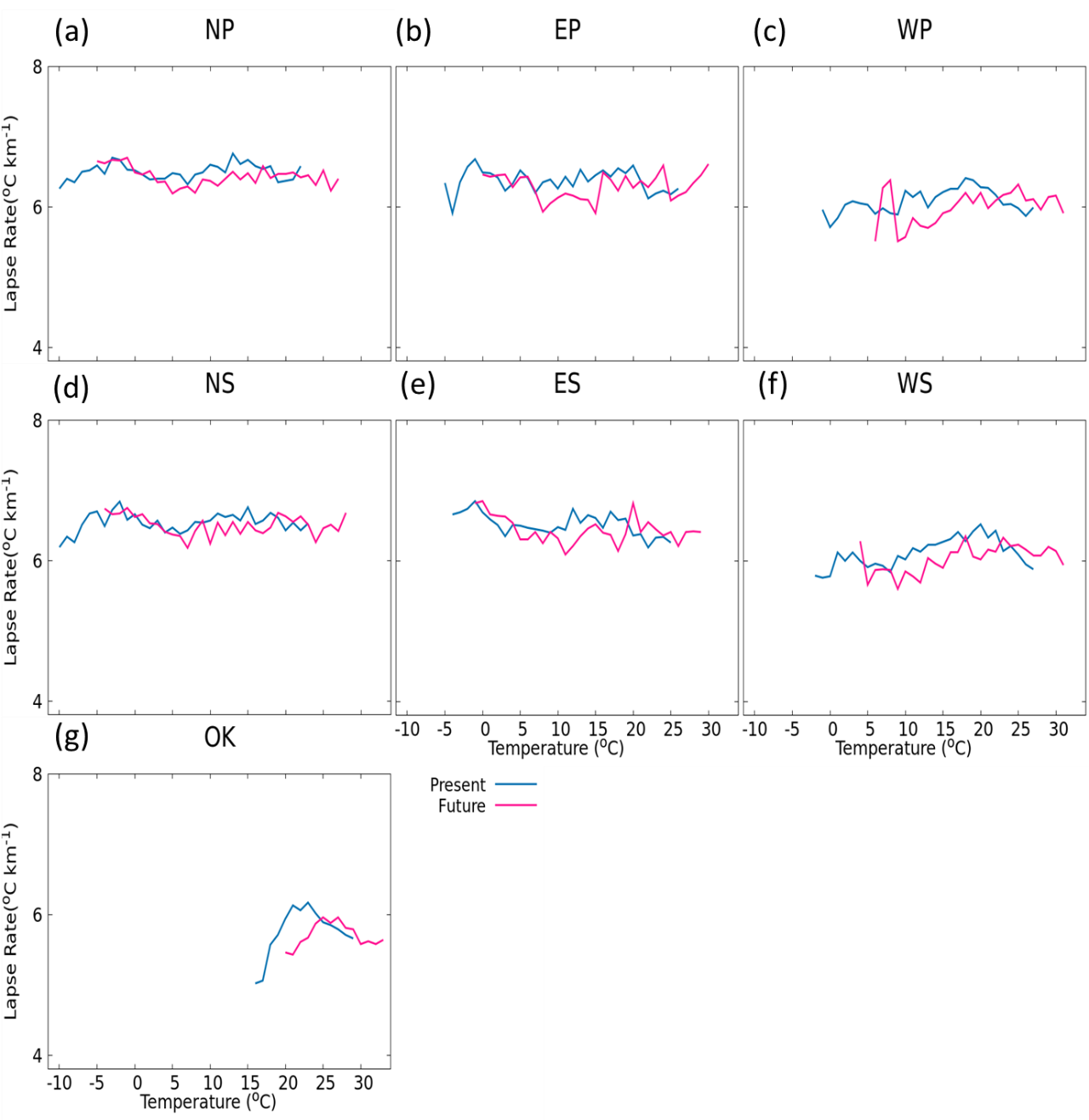
**

**Figure S4.** Lapse rate between 850 hPa and 500 hPa as a function of temperature during the wet events. The blue (magenta) color line represents the results from present (future) climate over North Japan (a, d), East Japan (b, e), West Japan (c, f) and Okinawa (g).


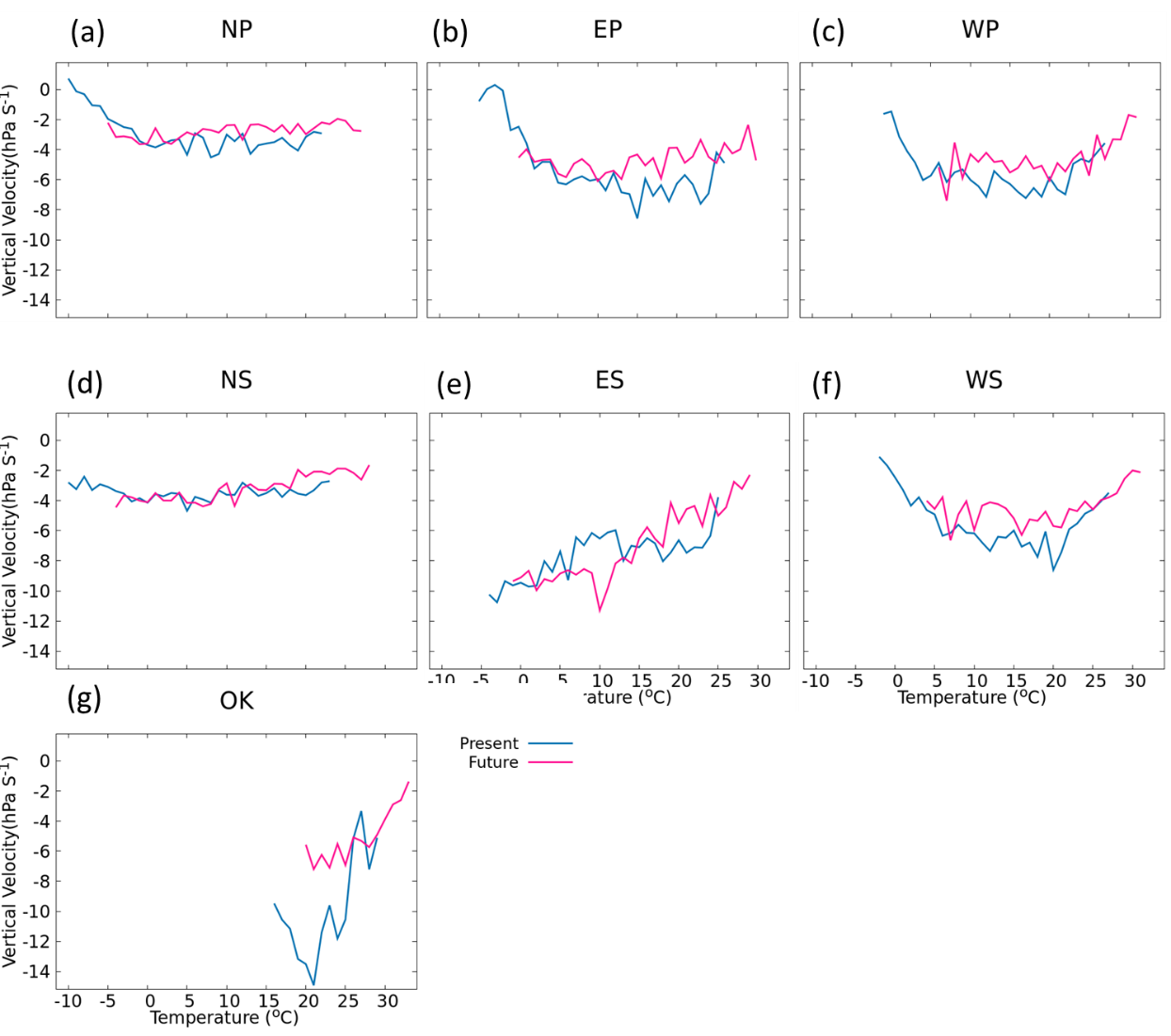


**Figure S5.** Vertical velocity at 700 hPa as a function of temperature during the wet events. The blue (magenta) color line represents the results from present (future) climate over North Japan (a, d), East Japan (b, e), West Japan (c, f) and Okinawa (g).

**Table S1**. Observed (APHRODITE) and simulated (d4PDF present climate) 99^th^ percentile precipitation for Okinawa with 1°C temperature bins and the bias.

| Temperature Bin (°C) | APHRODITE | d4PDF(Present) | Bias (%) |
| --- | --- | --- | --- |
| 17 | 49.10 | 30.31 | -38.26 |
| 18 | 62.34 | 38.10 | -38.88 |
| 19 | 73.55 | 49.19 | -33.12 |
| 20 | 77.62 | 57.65 | -25.73 |
| 21 | 91.37 | 66.63 | -27.08 |
| 22 | 90.07 | 70.52 | -21.70 |
| 23 | 102.20 | 71.93 | -29.61 |
| 24 | 111.53 | 73.38 | -34.21 |
| 25 | 143.65 | 77.41 | -46.11 |
| 26 | 147.34 | 82.10 | -44.27 |
